# The influence of school location and their children's nutritional model on the risk of obesity in Poland- pilot study

**DOI:** 10.3389/fnut.2025.1466065

**Published:** 2025-01-29

**Authors:** Łukasz Długoński, Anna Platta, Magdalena Skotnicka

**Affiliations:** ^1^Department of Commodity Science, Faculty of Health Sciences, Medical University of Gdansk, Gdansk, Poland; ^2^Faculty of Management and Quality Science, Gdynia Maritime University, Gdynia, Poland

**Keywords:** children, dietary habits, eating habits, obesity, rural-urban linkages

## Abstract

Problems with overweight and obesity during childhood and adolescence are associated with negative health effects that can impact a lifetime. Eating habits and lifestyles formed early in life influence our health in the future. These habits are difficult to change and often persist into adulthood. The aim of the study was to investigate the influence of residence and school attendance on the occurrence of childhood overweight and obesity. The survey, in the form of a questionnaire, was conducted among mothers or legal guardians of children attending public primary schools in the Pomeranian Voivodeship in Poland. A total of 515 individuals participated in the study. The questionnaire was prepared based on the Food Frequency Questionnaire (FFQ) and the Child Eating Behavior Questionnaire (CEBQ). The study showed that children living in rural areas are more prone to developing obesity and overweight. Among children living in rural areas, 46.89% are overweight, while in cities, 40.85% are overweight. Regarding children suffering from obesity, the percentage of those attending rural schools is 15.79%, while only 1.63% of obese children attend urban schools. Additionally, it was shown that children from rural areas more frequently exhibit poor eating habits and incorrect dietary patterns and behaviors, which mainly include eating lunch at school and simultaneously consuming nutrient-dense at home. Among children from rural areas, 74.07% consume sweets without restrictions, compared to only 25.93% of children from cities. The unrestricted consumption of salty snacks by children attending rural schools is as high as 75.64%, compared to 24.36% for children attending urban schools. The results of the current study indicate that residence and school attendance can determine the risk of developing overweight and obesity. Our findings show that children from rural areas have a high rate of obesity, highlighting the necessity to propose new solutions and tools to support proper nutrition, with particular emphasis on children from rural environments.

## 1 Introduction

Overweight and obesity during childhood and adolescence are associated with negative health consequences throughout life. Eating and other habits are formed early in life and continue until the end of life (1). The increasing prevalence of obesity in children is now a major public health problem worldwide, including Poland (2). The proportion of overweight and obese children has increased significantly in the last four decades, and obesity is described as one of the pandemics of the 21st century. Overweight and obesity in children also increase the risk of becoming overweight in adulthood, resulting in a higher risk of respiratory, metabolic and cardiovascular diseases compared to normal-weight children (3). Adequate nutrition plays an important role in maintaining overall health and wellbeing, and proper nutrition has a major impact on children's growth and development (4). Unhealthy behaviors that contribute to the development of obesity include an unhealthy diet, inappropriate eating habits, low levels of physical activity and a sedentary lifestyle (5). In the study, Trzcińska showed some signs of urbanization (urban-rural) diversity in terms of the prevalence of overweight and obesity in children. These phenomena generally occurred to a greater extent in rural environments than in urban ones (6). Witkowska and Lesiów. found that children living in the city (4.73%) were less obese than their peers from rural areas (6.42%). A large disproportion in obesity was noted between urban girls (4.17%) and their peers living in rural areas (8.62%) (7). In the countryside and in small towns research Hernández-Vásquez and Vargas-Fernández showed the largest increases in the prevalence of overweight and obesity in children in 2021 compared to 2019. They recorded the largest increases in the prevalence of eating disorders (8). Baran et al. showed that the place of residence has a significant impact on both the body fat content and the total water content in the body in children. Living in the city is associated with better body composition (9). Overweight and obesity in children and adolescents are increasing worldwide, although with different dynamics depending on the place of residence, with a significant increase currently observed in non-urban areas (10). The prevalence of obesity has increased in every country in the world, although there are regional differences in the prevalence of obesity (11). From the research of Sosnowska -Bielicz and Wrótniak it shows that obesity is diagnosed more often in children who live in the countryside (12). Panasiuk points to the increasing number of obese and lipid disorders in rural areas, which may result in an increase in the number of patients with type 2 diabetes and cardiovascular diseases in the near future (13). Numerous studies have been conducted worldwide on dietary habits and physical activity levels and their impact on overweight and obesity among school-aged children, highlighting the need for further research in this area. However, there is little research comparing obesity in children attending rural and urban schools, which may be relevant and indicate the need for a different approach to children's nutrition depending on where they live (14–16).

The aim of this study was to analyse the differences between urban and rural areas in the prevalence of overweight and obesity in primary school children and to identify which factors and dietary habits may determine the development of obesity.

## 2 Materials and methods

### 2.1 Study design

This study was a cross-sectional comparative study conducted in six public primary schools in the Pomeranian Voivodeship, Poland, from September 2022 to January 2023. The study targeted mothers or legal guardians of children attending these schools, with the goal of assessing the parental role in shaping children's eating habits. Participants completed a self-administered online survey. The study adhered to ethical standards, including anonymity and voluntary participation. Informed consent was provided on the survey's first page, in line with the Declaration of Helsinki (2000) and the European GDPR regulations (679/2016). Since the survey did not involve tracking sensitive personal data, ethics committee approval was not required. However, for the overall project analyzing behaviors, habits, and attitudes of children in the Pomeranian Voivodeship, approval was obtained from the bioethics committee (NKBBN/177/2021). Each questionnaire item was carefully formulated to minimize ambiguity, to further ensure data quality, the survey incorporated mandatory fields to prevent incomplete responses. Additionally, questions were formulated to be mutually exclusive to avoid overlapping categories and reduce potential biases.

### 2.2 Participants

The target population consisted of mothers or legal guardians of children attending public primary schools in Poland, aged 20 to over 45 years (x = 32.5). Most mothers are in the 25–29 age bracket, followed by 30–34 year olds. In terms of education, mothers with secondary and higher education dominate. On the other hand, in terms of work, most mothers are in white-collar jobs and fewer are in manual or non-working jobs (Table 1). Ultimately, 515 women participated after excluding incomplete responses (*n* = 7) and withdrawals (*n* = 18). Of the children in the study, only those who regularly attended physical education classes were included. For this reason, children who did not attend PE classes were not included in the study (*n* = 23). The basis for evaluating eating habits was derived from the questionnaire, which emphasized subjective assessments from the participants without direct anthropometric measurements of the children. Sampling involved contacting school administrators to invite eligible participants through student websites, social media, and electronic diaries. Participants had to be mothers or legal guardians of primary school children. Participants had to have access to the internet in order to complete the online survey. Data quality was managed by reviewing each response for completeness and logical consistency. Confounding factors were identified and managed based on predefined exclusion criteria.

**Table 1:** Characteristics of children and mother attending primary schools.

	**City school *n* = 306**	**Rural school *n* = 209**	**Total *n* = 515**
**Sex of the child**			
Girls	161	107	268
Boys	145	102	247
**Age of the child**			
Girls	(6–15)	(5–14)	(5–14)
Boys	(5–15)	(6–15)	(5–15)
**Body assessment of the child**	***n*** **/%**	***n*** **/%**	***n*** **/%**
Underweight body	65/21.24	18/8.61	83/16.12
Normal weight	111/36.27	60/28.71	171/33.20
Overweight	125/40.85	98/46.89	223/43.30
Obese	5/1.63	33/15.79	38/7.38
**Age of mother**	***n***	***n***	***n***
20–24	5	3	8
25–29	124	94	218
30–34	83	80	163
35–39	51	41	91
40–44	15	10	25
Above 45	5	5	10
**Education of mothers**			
Basic	15	10	25
Basic vocational	53	97	150
Secondary	121	74	195
Higher	101	44	145
**Type of work performed by mothers**			
Physical	46	99	145
Mental	153	37	190
Not working professionally	73	107	180

### 2.3 Data collection

Data was collected using an anonymous online survey developed in Google Forms and distributed through school administrators. The survey link was shared via student websites, social media and the Librus electronic diary to reach mothers or guardians of children in the six targeted primary schools. Participation was voluntary and respondents were assured of anonymity and confidentiality.

The survey consisted of 15 closed-ended questions that collected basic demographic information, mothers' self-reported dietary habits, and their perceptions of their children's dietary behavior, which we refer to as the “children's dietary model.” In this context, the “child feeding model” represents mothers' subjective assessments of their children's eating patterns, meal frequency and food choices as understood by the mothers. The variables studied included demographic variables: mother's age, area of residence (urban/rural). Maternal dietary habits and behavior based on questions from the Food Frequency Questionnaire (FFQ). Children's eating behavior assessed by maternal perception using items adapted from the Child Eating Behavior Questionnaire (CEBQ). Children's weight status assessment: mothers categorized their child's weight status as underweight, normal weight, overweight or obese, providing insight into maternal perceptions of children's physical development. No personal identifiers were collected to ensure the anonymity of respondents. Participants received no compensation for completing the survey.

### 2.4 Data analysis

The analysis was based on Statistica 12.0 Advanced Pack with Plus Kit, PQStat 1.8.0.438 and Excel 2007. Statistics on qualitative variables were used because they occur in research (chi-squared test, correspondence analysis). The chi-squared test is a statistical tool used to test the correspondence between the empirical and theoretical distributions. It was checked whether there is a relationship between two nominal variables and whether the frequency of occurrence of certain phenomena is in line with theoretical expectations. The study also performed correspondence analysis to show possible relationships between nominal variables. The analysis of correspondence allowed to identify patterns in the data and determine which variables are correlated with each other and which are independent.

## 3 Results

### 3.1 Characteristics of children's silhouettes

This study analyzed the responses of 515 mothers whose children attend public primary schools in Poland. The survey consisted of 15 questions about the behaviors and eating habits of mothers and their children. The study was conducted by differentiating responses according to where children go to school.

**Figure 1:**
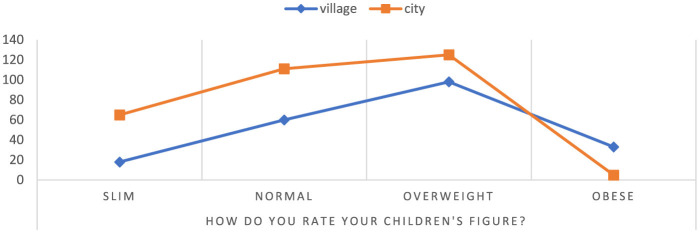
Assessment of the child's silhouette from the perspective of mothers. Chi^∧^2 Pearsona: 492,012, df = 3, *p* = 0.000000, statistically significant differences *p* < 0.05.

In the study, women described the profiles of their children based on the knowledge available to them. They rated weight status in one of four categories: underweight, normal, overweight, obese (Figure 1). Among people living in rural areas, most respondents assess their figure as overweight-−46.89%, and the least as underweight-−8.61%. In the city, on the other hand, most people assess their figure as **normal weight**-−36.27%, and the least as obese-−1.63%. The results also shows the number of respondents in each weight status category in individual places of residence.

### 3.2 Determinants shaping overweight and obesity among children

Overweight and obesity are harmful to health, which are increasingly increasing the number of children around the world. A number of factors have been found to be these factors, including genetic, causal and behavioral causes. Among children who are overweight and obese, due to the risk of diseases. This study aims to identify and analyze the specific factors influencing overweight and obesity in children, examining how elements such as diet contribute to changes in weight and overall health outcomes. The goal of many scientific studies is to understand the determinants that shape overweight and obesity among children. In this context, this research focuses on the factors that influence children's weight gain and the **hearty**. Table 2 presents the list of questions in the questionnaire and their statistical significance. The significant value of the graph refers to how similar the results obtained in the result are. In the inquiry questionnaire, provide 15 questions, and each of them has its own statistical significance. To calculate individual statistics, the chi^2^ test was used at significance level *p* < 0.05. Our research showed that the majority of respondents (93.30% rural and 88.24% urban) said that it is the parent who decides on the daily menu of their child (*p* = 0.06). In addition, the majority declared that they cook dinners at home (94.50% rural and 93.32% city). At the same time, it was noticed that most children willingly eat meals prepared by their mothers (97.52% rural and 95.44% urban). Based on the survey declaration, as many as 98.85% of mothers living in rural areas and 94.55% of mothers living in cities, regularly prepare meals together with their children. For this study, no significant relationship was found between the groups, meaning that the meal preparation model in this area does not differ depending on the place of residence (*p* = 0.06). In the countryside as well as in the city, most families eat at the common table. Among the surveyed rural families, as many as 97.65% eat meals together, while among families living in the city, similar customs are practiced by 93.45% of the surveyed families. The percentage of children attending rural schools who do not eat fruit at all is 5.13%, while in the case of children attending urban schools it is 7.07%. Among the surveyed group of rural students, as many as 45.20% reach for fruit every day. Similar results were recorded among children attending urban schools 41.80%. In the group of respondents from rural areas, 47.37% answered that their child receives money to school in order to use it for any snack, while in the group of respondents from the city 49.02%. In this case, no statistically significant differences were found between children from the city and the countryside (*p* = 0.71). In Polish educational institutions, one of the available ways to spend these funds are snack vending machines, which offer a variety of food products, including sweets, salty snacks and sweetened beverages. Consumption of these products can contribute to weight gain and health problems in children. Unfortunately, due to the inability to leave the school grounds to buy healthy snacks, children often choose unhealthy, processed foods. It can therefore be concluded that the money received from parents for snacks encourages children to eat meals that are unfavorable to health. 47.80% of rural school children and 52.50% of urban school children eat sweets between meals every day, while 31.10% of rural children and 29.80% of urban children eat salty snacks between meals every day. According to the conducted research, the amount of fluids drunk by children from rural and urban schools is similar. No statistical difference was found between the studied groups of children (*p* = 0.06). 26.55% of children attending rural schools and 19.78% of those attending urban schools drank 0.5 L of water daily. These factors can determine the promotion of obesity. Nevertheless, children attending rural schools are overweight. The phenomenon associated with higher rates of obesity among rural children compared to urban children may be related to differences in many factors, such as lifestyle, diet and environment.

**Table 2:** Questionnaire questions and their statistical significance.

**No**.	**Question**	***p***
1	Who decides about your child's daily menu?	0.06
2	Does your child eat lunch at school?	0.00
3	Does your child eat dinner at home?	0.00
4	Do you cook dinners at home?	0.06
5	Does your child enjoy eating meals prepared by you?	0.09
6	Do you prepare meals together with your child?	0.06
7	Do you have lunch with your child at the same table?	0.07
8	How often does your child eat sweets?	0.00
9	How often does your child eat salty snacks?	0.00
10	How often does your child drink sweetened beverages?	0.00
11	How often does your child eat fruit?	0.09
12	Does your child receive money for school to use for any snack?	0.71
13	How many meals a day does your child eat?	0.00
14	What foods does your child usually eat between meals?	0.07
15	How much fluid does your child usually drink during the day?	0.06

Statistically significant differences *p* < 0.05.

### 3.3 Differences in the model of nutrition for children from rural and urban areas

In Poland, school canteens are a popular solution for parents who want to provide their children with healthy and nutritious meals during the day. However, as statistics show, there is a difference in the use of this opportunity between children from the city and the countryside, where a higher percentage of children in the city eat lunch at school. For the entire study group, the percentage of children eating lunch at school is 71.07%. Children from urban areas more frequently take advantage of school lunches (78.76%) compared to children from rural areas. The percentage of rural children eating lunch at school is relatively low, at 59.81%.

**Figure 2:**
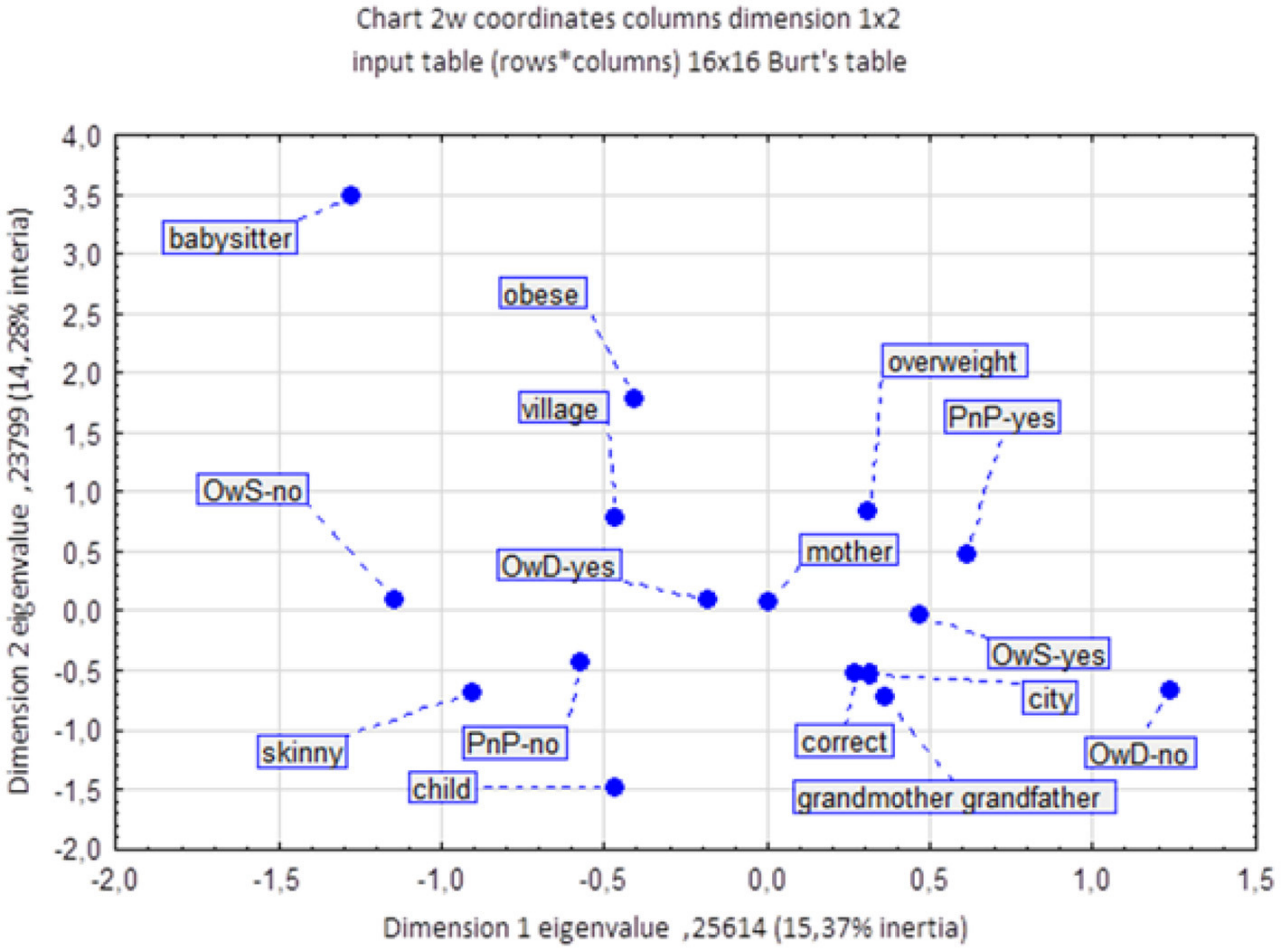
The influence of determinants on the occurrence of obesity in children. OwD, lunches at home; OwS, lunches at school; PnP, money for snacks.

**Figure 3:**
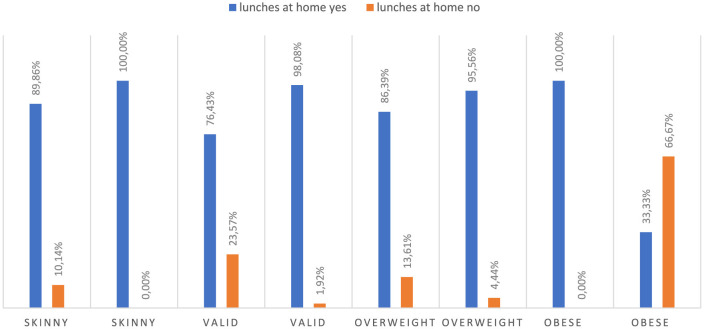
Lunches at school vs. lunches eaten at home. Chi^∧^2 Pearsona: 53.8500, df = 10, *p* = 0.000000, statistically significant differences *p* < 0.05.

Due to the fact that a significant portion of children from rural areas do not eat lunch at school, the results show that 93.30% of rural children eat lunch at home. Among urban children, 83.33% eat lunch at home. Based on the results, it can be concluded that there is a statistically significant difference between the percentage of children eating lunch at home in rural and urban areas (*p* = 0.000823). Additionally, in our study group, there are children who eat two lunches, one at school and one at home. However, the correspondence analysis (Figure 2) illustrates the associations between various factors related to childhood obesity. The analysis reveals that place of residence plays a significant role in the prevalence of obesity among children. Children attending rural schools (represented by the “village” category) are more likely to be obese than those living in urban areas. This suggests that rural environments may be associated with factors that contribute to a higher risk of obesity, possibly due to lifestyle or dietary habits prevalent in those areas. Another notable factor influencing childhood obesity is the role of the mother in dietary habits. The proximity of “mother” to “overweight” and “obese” on the chart indicates a strong association between a child's weight status and maternal influence on diet, particularly regarding meals prepared at home (“OwD-yes”). This suggests that mothers, as primary decision-makers in meal preparation, significantly impact children's nutritional intake, which can contribute to overweight and obesity. The chart also highlights the impact of school meals and snacks on children's weight status. Children who do not consume meals at school (“OwS-no”) are more closely associated with “underweight” and “child,” suggesting that those who bring meals from home or eat at home may have different dietary outcomes. Additionally, children who receive money for snacks (“PnP-yes”) are associated with being “overweight,” indicating that access to funds for potentially unhealthy snacks increases the likelihood of overweight. The analysis further examines the role of other caregivers, such as babysitters, grandmothers, and grandfathers. While these caregivers are positioned separately from the “mother” category, their association with obesity is weaker. Nevertheless, their positions suggest a potential, albeit less pronounced, influence on the dietary choices and weight status of the children they care for. In conclusion, the correspondence analysis confirms that childhood obesity is a complex issue influenced by a combination of factors, including the child's environment, the primary caregiver's influence, and access to snacks at school. These results underscore the multifaceted nature of childhood obesity, shaped by place of residence, meal source, and caregiver roles in determining dietary habits and weight outcomes. The study revealed a difference in dietary preferences among different weight status. Children with obesity were observed to more frequently consume school meals compared to their peers (Figure 3). Overweight children are more likely to choose school meals, whereas underweight children prefer home-cooked meals. These results indicate a relationship between weight status and dietary preferences. It is worth noting that children who eat lunch at school and then return home often eat a second meal, as this is the preference for most overweight children. A noteworthy finding from this study is that overweight children are more likely to have lunch at school, with 13.61% of overweight children not eating lunch at home. For many of these children, having lunch at school often leads to eating a second meal when they return home, contributing to an increase in overall calorie intake. This pattern of consuming both a school lunch and a second meal at home appears to be a prevalent behavior among overweight children, potentially influencing their weight gain. By contrast, underweight children overwhelmingly prefer home-cooked lunches, with 89.86% of them eating lunch at home and therefore not engaging in this “double meal” pattern. This suggests that a single, home-prepared meal might help maintain a healthier weight. A statistically significant relationship (*p* = 0.000000) was found between the occurrence of obesity and eating lunches both at school and at home in 1 day.

**Figure 4:**
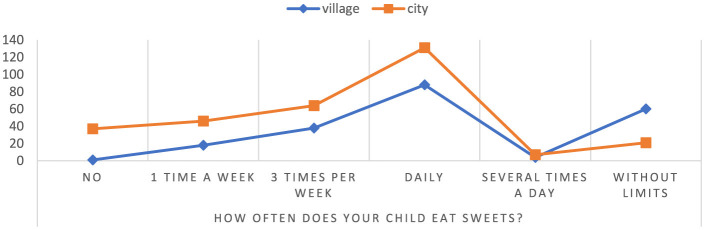
The frequency of consumption of sweets by children. Chi^∧^2 Pearsona: 65.0597, df = 5, *p* = 0.000000, statistically significant differences *p* < 0.05.

The results of the study showed that children attending schools in rural areas consume sweets more often than children living in cities regardless of weight status (Figure 4). Among children from rural areas, as much as 74.07% consume sweets without restrictions, while among children from cities it is only 25.93%. Additionally, 40.18% of all children in rural areas and 59.82% of children in urban schools consume sweets every day. Only 37 mothers said that their children attending city schools did not eat sweets at all, while one mother of a child attending a rural school said that her child did not eat sweets at all. The presented results indicate a significant difference in the consumption of sweets between children from different residential areas (*p* = 0.000000). They also highlight the fact that children from rural areas have a greater tendency to consume sweets without restrictions compared to children from cities. Moreover, the study results point to a considerable number of children who consume sweets regardless of their place of residence (children from urban areas-−269, children from rural areas-−208).

**Figure 5:**
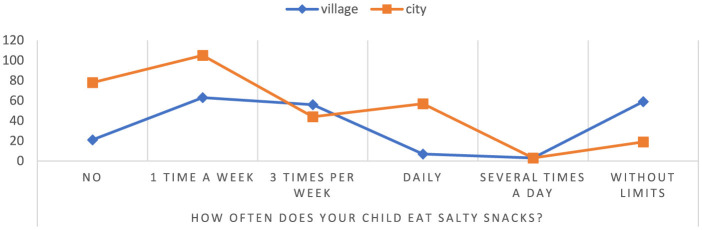
The frequency of consumption of salty snacks by children. Chi^∧^2 Pearsona: 89.2290, df = 5, *p* = 0.00000, statistically significant differences *p* < 0.05.

Based on the conducted research, it has been shown that the consumption of salty snacks without restrictions by children attending rural schools is as high as 75.64% (children attending schools 24.36%) (Figure 5). In addition, in the group of children attending urban schools, a lack of salty snacks was observed to a high degree, as much as 78.79%, compared to only 21.21% in the group of children from rural schools. The results indicate a significant difference in salty snack consumption between rural and urban school children *p* = 0.00000. The results point to potential differences between urban and rural school children with respect to eating habits and lifestyle. This may be due to differences in the availability and quality of food in cities and villages, as well as differences in lifestyles such as physical activity.

**Figure 6:**
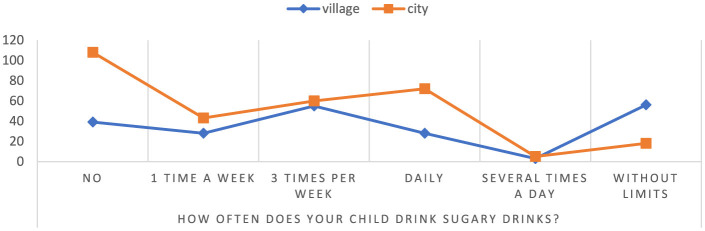
Frequency of consumption of sweetened beverages by children. Chi^∧^2 Pearsona: 58.9698, df = 5, *p* = 0.000000, statistically significant differences *p* < 0.05.

According to the study, it was found that the percentage of children attending rural schools consuming sweetened beverages without restrictions is 75.68%, while the percentage of children from urban schools is 24.32% (Figure 6). Additionally, among children studying in urban schools, there was a lack of sweetened beverages consumption in 73.47%, compared with a rate of 26.53% among children from rural schools. These results suggest that children attending rural schools may have a higher risk of health consequences associated with excessive consumption of sweetened beverages, such as diabetes, overweight or cardiovascular disease *p* = 0.000000. On the other hand, children from urban schools tend to avoid sweetened beverages, which may indicate greater health awareness in this group, but it is worth paying attention to whether they do not reach for other unhealthy snacks instead.

**Figure 7:**
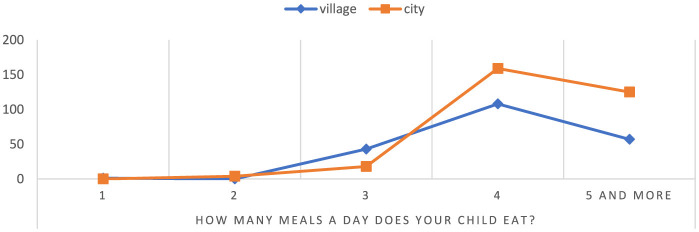
Number of meals consumed per day by children. Chi^∧^2 Pearsona: 33.3057, df = 4, *p* = 0.000001, statistically significant differences *p* < 0.05.

According to statistics, children attending rural schools eat an average of five meals a day at a rate of 31.32%, while in urban schools it is 68.68% (Figure 7). In rural areas, 70.49% of children eat three meals a day, while in cities it is 29.51% of the child population. The results suggest significant differences in eating habits between children from different backgrounds, which may have an impact on their overall health. On average, children attending urban schools eat fewer meals per day than children in rural areas *p* = 0.000001. In addition, most urban children eat only three meals a day, which means they may tend to eat larger portions during these three meals, which can affect their weight and health. Children in urban areas eat more meals a day, which may suggest that they have better eating habits and greater access to food, which can affect their overall health and development.

There are significant differences in eating habits between children in rural and urban areas, which may be the result of differences in access to healthy food, lifestyle and other factors affecting nutrition.

## 4 Discussion

The results of our study demonstrate distinct differences in the eating behaviors and weight status of children from urban and rural areas in Poland. Children from urban settings tend to have more balanced body composition, with lower rates of overweight and obesity, whereas children in rural areas show a higher prevalence of body weight disorders, including overweight and obesity. Research by Baran et al. shows that there are significant differences between the weight status of children living in cities and children living in small towns and in the countryside. Living in the city results in more favorable proportions between different elements of the body. This study referred to the percentage of body fat in children (17, 18). Similarly, a study by Bergier et al. found that place of residence influences obesity occurrence, with BMI levels being less favorable among rural residents (19). In the rural group, disorders in body weight, including overweight and obesity, were observed (20). Additionally, Sygit et al. noted improper eating habits in the rural population, with many respondents consuming only 1–2 meals per day and giving little attention to eating breakfast or a second meal. Fruits and vegetables were often omitted from the diet, while sweets were highly consumed. Our research similarly found that most children from villages eat only three meals a day, possibly leading to larger portions per meal (21). According to the study of Cipora et al., children living in the countryside follow the principles of rational eating, but it cannot be concluded that the behaviors are correct. Children in rural areas require health education in nutrition and physical activity. As one of the many mistakes mentioned is not taking care of eating and snacking between meals. Regardless of where children attend school eat sweets between meals. It is recommended to introduce nutritional prevention and promote a healthy lifestyle (22). According to our findings, the amount of fluids drunk by children from rural and urban schools is similar. 26.55% of children attending rural schools and 19.78% of those attending urban schools drank 0.5l of water daily. Eating and drinking habits developed during childhood are an integral part of a healthy lifestyle. Daniels and Popkin suggests water may play a key role in reducing energy intake, which in turn contributes to the prevention of obesity (23). Story et al. noticed that school meals eaten by children may contribute to obesity. This was also confirmed by our research (24). Kirkpatrick et al. showed that children in rural and suburban areas consumed more junk foods, sweetened beverages, and sweets. Low farm income had the greatest impact on the prevalence of overweight and obesity in rural areas (25). Bracale found that children's attitudes toward fruit and vegetable consumption are influenced by parental diets and lifestyles. In our study, the percentage of children who do not eat fruit was similar in both rural and urban schools. Family size and parental education also correlated with overweight risk among urban children (26), while our findings showed no impact of shared family meals on obesity. Vik et al. found a negative correlation between eating breakfast together and children's weight (27). However, our study indicates that mothers often shape children's diet choices, potentially contributing to childhood overweight, as children typically enjoy meals prepared by their mothers. Although strategies for preventing childhood obesity are often directed at parents, children increasingly spend time with informal caregivers, especially grandparents. Studies have shown that grandparental care can introduce less healthy eating practices, sometimes using food to regulate emotions (28). Rhodes et al. found that grandmothers and mothers had the greatest impact on food choices. Grandparents and children had a strong bond and relationship that influenced eating behavior (29). Grandparents' eating practices, such as eating a lot of calorie, processed and fatty foods, can be a risk factor for obesity in their offspring because they influence the formation of eating habits and taste preferences already in childhood and youth, which can influence further food choices and lifestyle. In addition, lifestyle changes toward more sedentary and less physical activity also contribute to the development of obesity. In addition, hereditary predispositions to obesity may also play a role in this process, and grandparents' eating practices may work synergistically with this propensity (30). The results of the Farrow study indicate that children's eating behavior can be significantly shaped by their grandparents, which in turn can affect the eating habits of grandchildren (31). Grandparents have a significant impact on the lives of their families as well as their communities, although the type of care they provide, the intensity and their role as primary or secondary caregivers varies. Children living with grandparents are characterized by a diverse family structure and level of economic wellbeing, which can affect the health of family members in different ways (32). Research An et al. showed that children who were under the care of grandparents were at increased risk of overweight and obesity (33). Jingxiong et al. played an important role of grandparents and their influence on children's nutrition and eating habits. Grandparents believed that overweight children would be more developed and tall. Children were urged to eat more food by grandparents who used food as an educational and emotional tool (34). Children who have the means to buy food in school vending machines show a higher weight, which was also confirmed in our research (35). In the Kujawska-Pac et al. study, the most exposed goods in school shops and vending machines are still drinks and salty snacks, bars, wafers, juices and chocolate bars. Such processed products can contribute to childhood obesity (36). In research, Zielińska et al. proved that the most frequently purchased products at school include sweets (55%), sweet rolls (24%) and other products (19%). This state of affairs largely contributes to the occurrence of overweight and obesity in children (37). Most of the groceries you buy in stores are processed and inexpensive. Improving the quality of food in school shops and vendors could improve the overall diet of teenagers (38). Afr proved that children preferred to spend the money they received from their parents to buy food in the school store. Because of the price, convenience, peer influence and popularity. Cookies and salty snacks were the first choice of children (39). Our study found no significant differences in family mealtime practices across urban and rural settings. Watts et al. underscore the long-term impact of parental practices, including family meals, on children's habits into adulthood, suggesting that dining together may reduce obesity risk by promoting healthy habits and family bonds (40).

The findings of Sun et al. suggest the need to develop specific nutritional strategies to prevent the occurrence of overweight and obesity in children (41). Pereira and Oliveira showed that most dietary actions aimed at combating childhood obesity focus on an individual, education-based approach, while less attention is paid to changes in the environment that may promote healthier behaviors. Unfortunately, most of these activities do not bring the expected effects in the form of reducing obesity in children. It seems that the best approach to combating the childhood obesity epidemic is to create environments conducive to healthy eating habits and physical activity. Complex interventions focusing on environmental change and the empowerment of individuals and communities, including families, as well as macroeconomic policy changes, have the potential to effectively tackle childhood obesity without increasing socio-economic inequalities. Key factors in avoiding overweight and obesity in children are changing parents' attitudes toward healthy eating. Children should be provided with knowledge about healthy food. Some of the many barriers are insufficient knowledge of dietary recommendations, lack of knowledge about healthy recipes and choices about eating out. Changing food procedures into family eating and promoting healthy eating among children is a good way to prevent childhood obesity. Involving children in the preparation of meals can also bring tangible benefits (42). Public health and nutrition education and lifestyle actions are required to promote and support healthy eating among children. Such actions can bring the expected results in the health of future generations (43). Interdisciplinary intervention programs can provide a real opportunity to promote healthy lifestyles, which in turn would foster lasting change in this area. The main objective of preventing childhood obesity should be to create a health education program, especially in the context of adequate nutrition in kindergartens and schools, and to provide the infrastructure for its implementation. It is necessary to increase the physical activity of children and their families by supporting health-promoting movements and organizations in schools and other educational institutions. This, in turn, leads to a lack of knowledge in society about the role of nutrition and physical activity in maintaining health (44). Gülü et al. found that children living in rural areas were much more likely to be overweight and obese. Obese children had higher levels of food addiction. Another proven cause was a lower level of physical activity. In order to prevent overweight and obesity in children, it is suggested to reduce food dependence and increase the level of physical activity. In most cases, the development of obesity in children is associated with unhealthy eating habits and lack of physical activity. For this reason, health programs implemented in schools that aim to prevent overweight and obesity should focus mainly on these two risk factors that can be changed (45). The prevalence of obesity is higher among children living in rural areas than in urban areas. What was surprising in the study of Tambalis et al. was that children living in rural areas had higher levels of self-reported level of physical activity (46). The results of the study indicate an increase in the number of overweight rural children, which indicate genetic, environmental, socio-economic risk and the prevalence of obesity in parents and their income as the cause (47). Rural areas experience problematic health effects such as obesity and related chronic diseases. The study by Kaczynski et al. emphasizes that efforts are needed to improve rural environments. Stresses the special role of higher education, policies and initiatives. Rural areas are more conducive to the occurrence of obesity (48). Koester et al. in their research indicate the introduction of training and educational programs as prevention of obesity in children from rural areas (49).

However, some limitations of this study should be noted. They were conducted using a questionnaire shared with mothers of children attending urban and rural schools. In the questionnaire, women were asked to rate the physique and behavior of their children, which can lead to subjective answers, which is quite often observed in this type of research. The survey was only carried out in the northern part of Poland on a representative sample, which may be a limitation. Our study is only a fragment of a wider study on the problem of overweight and obesity in children. Further research is needed to identify the factors contributing to the dynamic prevalence of overweight and obesity in children, especially in Poland. Determining the causes of overweight and obesity among children can effectively help develop successful nutritional and educational strategies. Our study focused on the impact of environmental parameters, particularly in the context of residence. The identified differences in the habits and behaviors of children living in cities and villages highlight the need for a different nutritional program for children. It is possible that the currently implemented educational strategies for children and their parents in rural areas are poorly chosen and improperly constructed, as they have not yielded the desired results for years. Therefore, our study is crucial as it confirms and highlights the urgent need to introduce new tools for managing children's nutrition, especially in rural environments.

## 5 Conclusions

The results of pilot studies suggest that the place of residence and school attendance may influence the risk of developing overweight and obesity in children. Our findings show that children from rural areas in the Pomeranian Voivodeship have a high rate of obesity. Among the factors influencing the development of overweight and obesity in children are eating lunch at school while also consuming nutritious meals at home, eating sweets, salty snacks, and drinking sweetened beverages without restrictions. The survey results indicate that responses from mothers of children from rural areas more frequently pointed to dietary errors that may promote obesity. The research highlights the necessity of extending the analysis to other regions of Poland to effectively implement educational strategies, particularly in rural areas.

## Data Availability

The raw data supporting the conclusions of this article will be made available by the authors, without undue reservation.
